# Exact correspondence between walk in nucleotide and protein sequence spaces

**DOI:** 10.1371/journal.pone.0182525

**Published:** 2017-08-11

**Authors:** Dmitry N. Ivankov

**Affiliations:** 1 Laboratory of Evolutionary Genomics, Bioinformatics and Genomics Programme, Centre for Genomic Regulation (CRG), Barcelona, Spain; 2 Universitat Pompeu Fabra (UPF), Barcelona, Spain; 3 Laboratory of Protein Physics, Institute of Protein Research of the Russian Academy of Sciences, Pushchino, Moscow region, Russia; Harbin Institute of Technology Shenzhen Graduate School, CHINA

## Abstract

In the course of evolution, genes traverse the nucleotide sequence space, which translates to a trajectory of changes in the protein sequence in protein sequence space. The correspondence between regions of the nucleotide and protein sequence spaces is understood in general but not in detail. One of the unexplored questions is how many sequences a protein can reach with a certain number of nucleotide substitutions in its gene sequence. Here I propose an algorithm to calculate the volume of protein sequence space accessible to a given protein sequence as a function of the number of nucleotide substitutions made in the protein-coding sequence. The algorithm utilizes the power of the dynamic programming approach, and makes all calculations within a couple of seconds on a desktop computer. I apply the algorithm to green fluorescence protein, and get the number of sequences four times higher than estimated before. However, taking into account the astronomically huge size of the protein sequence space, the previous estimate can be considered as acceptable as an order of magnitude estimation. The proposed algorithm has practical applications in the study of evolutionary trajectories in sequence space.

## Introduction

Evolution can be envisioned as a “walk” of biological sequences in a sequence space [1]. Protein evolution has been modelled in two ways. As an approximation, protein sequences accept amino acid substitutions in the protein sequence space [2,3], which is a substantial simplification that ignores the properties of the genetic table. In a more realistic approach, protein-coding nucleotide sequences walk in the nucleotide sequence space, while their translation tracks the corresponding walk of protein sequences in protein sequence space [4].

To understand protein evolution, it is crucial to explore the correspondence between the protein and nucleotide sequence spaces. The simplest example of such correspondence is that 4^3*n*^ nucleotide sequences map to 20^*n*^ protein sequences for the set of *n*-residue proteins. However, other relationships are less trivial to establish, so they may require algorithmic or approximate solution because brute-force calculations could be prohibitively long. For example, how the size of the protein sequence space accessible to a given protein sequence increases with the number of nucleotide substitutions introduced in the protein-coding sequence? This task emerged in the experimental characterization of local fitness landscape for green fluorescence protein (GFP) [5], where Sarkisyan *et al*. were interested in the fraction of sequence space sampled by GFP mutants. Sarkisyan *et al*. estimated this fraction by counting the fraction of missense substitutions from among all nucleotide substitutions for specific codons and taking the average across all codons coding for the same amino acid [5]. However, the accuracy of this approximation has not been tested and for future studies it may be important to find an exact solution and to compare it with the approximate one.

Here, I present an efficient algorithm that accurately calculates how the size of accessible protein sequence space depends on the number of nucleotide substitutions introduced in the protein-coding sequence. The algorithm is based on dynamic programming, a powerful technique for solving optimization tasks of a combinatorial nature [6]. The presented algorithm can be used to study protein sequence evolution and to solve related tasks in computational biology.

## Algorithm

As a core procedure, the presented algorithm calculates the increment of the protein sequence space accessible to a protein sequence as a function of the number of nucleotide substitutions introduced in the protein-coding sequence. In other words, the algorithm sets the given protein sequence as a reference point, divides the protein sequence space around the protein sequence into regions accessible by one, two, etc. nucleotide substitutions, and calculates the volume of each region. These regions do not overlap because a protein sequence is counted when it is obtained by the minimal number of nucleotide substitutions.

**Fig 1:**
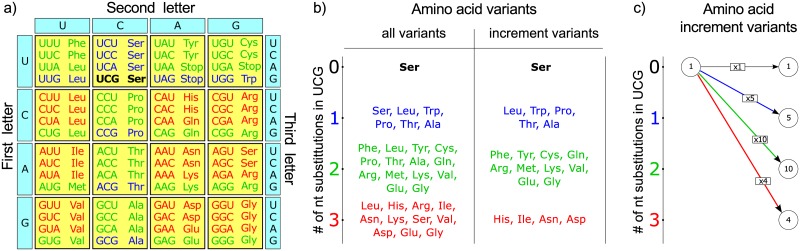
Consideration of the serine-coded UCG codon. (a) The standard genetic code table with codons colored by distance from the considered UCG codon: UCG codon itself is colored black; codons at the distance of one, two and three nucleotide substitutions, are colored by blue, green and red, respectively. (b) The list of amino acids that can be obtained from serine UCG codon by zero (black), one (blue), two (green), and three (red) nucleotide substitutions. On the left all amino acid variants are given, while on the right only variants are given that contribute to the increment of the protein sequence space. (c) The graph representation of the number of possible amino acid variants when mutating UCG codon. Black, blue, green, and red arrows correspond to zero, one, two, and three nucleotide substitutions, multiplying the previously available number of amino acid variants (here one, left circle) by one, five, ten, and four variants, respectively.

First, I inspected the case of one codon. Fig 1 illustrates this using the serine codon UCG as an example. In the current form the algorithm assumes that a nucleotide site can accept at most one substitution (this is a reasonable assumption if the number of nucleotide substitutions is much less than the nucleotide sequence length). Fig 1a shows the standard genetic code table where codons are colored by their distance to UCG. After one, two, and three nucleotide substitutions, the codon UCG can mutate to nine (blue), 27 (green) and 27 (red) codons, respectively. At the same time, the UCG-coded serine (Fig 1a and 1b, left panel) mutates to six (blue), fourteen (green) and eleven (red) amino acid residues.

Notice that most residues can appear after different number of nucleotide substitutions introduced to UCG (Fig 1b, left). For example, leucine can appear after one or two substitutions, while serine itself can appear after zero, one, or three, but not two substitutions (Fig 1a and 1b, left). However, we need to leave the amino acid only once, when it is realized by the minimal number of nucleotide substitutions, because we are interested only in the newly accessible amino acid variants. Indeed, if an amino acid variant has already been realized with fewer number of nucleotide substitutions, it has already been counted as a part of one of the previous increments. After removing duplicates, for zero, one, two, and three nucleotide substitutions in UCG-coded serine we obtain, respectively, one, five, ten, and four newly accessible amino acids (Fig 1b, right panel). I will denote these numbers of the newly accessible amino acid variants as *M*_*i*, *codon*_, where *i* (*i* = 0, 1, 2, 3) is the number of nucleotide substitutions for a given *codon*:

$$M_{0,\;UCG}\;=\;1,M_{1,\;UCG}\;=\;5,M_{2,\;UCG}\;=\;10,\text{ and }M_{3,\;UCG}\;=\;4.$$

The values *M*_*i*, *codon*_ (*i* = 0, 1, 2, 3) are calculated for each codon directly from a genetic code table.

Now let me consider the general case of several codons. If I add the UCG codon to the sequence of codons having already some number of amino acid variants, it will increase this number by the factor of *M*_*i*, *UCG*_ depending on the number of nucleotide substitutions *i* additionally introduced in the UCG codon. Fig 1c illustrates this for the case when the preceding sequence has one amino acid variant. Generally, the number of amino acid sequences *N*_*i*, *j*_, comprising the increment of protein sequence space for *i* nucleotide substitutions introduced in the sequence of *j* codons can be calculated recursively:

$$N_{i,j}=\sum_{k=0}^{\text{min}\{3,i\}}N_{i-k,j-1}\cdot M_{k,codon_{j}}.$$ (1)

**Fig 2:**
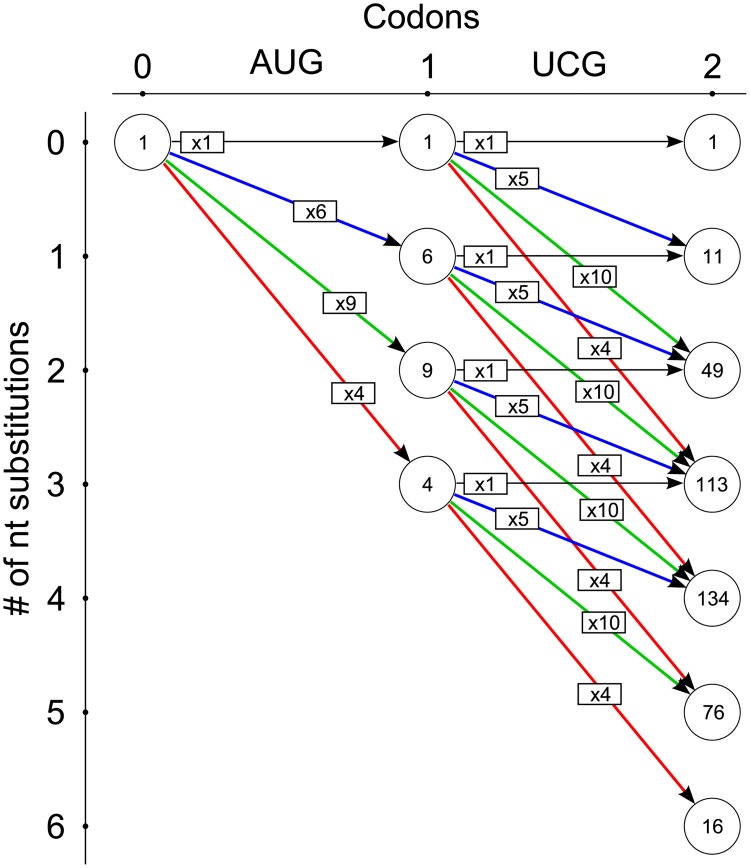
The illustration of dynamic programming procedure. The example of nucleotide sequence AUG UCG coding for Met-Ser amino acid sequence is considered. The colors of the arrows denote the same as in the Fig 1c.

Here *N*_*i-k*, *j-1*_ is the size of protein sequence space increment for *i—k* nucleotide substitutions introduced in the prefix subsequence of length *j*– 1. Clearly, *N*_*0*, *0*_ = 1 because only one sequence exists of zero length with zero substitutions, the empty sequence. The algorithm corresponding to Eq 1 is illustrated by Fig 2 for an example of the short nucleotide sequence AUG UCG coding for dipeptide MS.

Now, it is straightforward to go from the incremental characteristics to the integral one. That is, the volume of the protein sequence space *L*_*p*, *j*_ accessible for a protein sequence after introducing a given number of nucleotide substitutions *p*, equals to:

$$L_{p,j}=\sum_{i=0}^{p}N_{i,j}.$$ (2)

It sums up all increment volumes obtained by up to *p* nucleotide substitutions.

Sometimes, it may be necessary to calculate the protein sequence space volume when keeping constant the starting (or any other) codon. In that case, for starting codon only zero mutations are possible:

$$M_{0,\;start}\;=\;1,\text{ and }M_{1,\;start}\;=\;M_{2,\;start}\;=\;M_{3,\;start}\;=\;0.$$

The exploration of the required time and space shows that for a given number of nucleotide substitutions *p* the algorithm complexity is *O(p R)* both in time and space, where *R* is the number of codons in protein, i.e., the number of amino acid residues. In the worst scenario, a user can ask to calculate the number of nucleotide substitutions *p = 3R*, and the complexity becomes *O(R*^*2*^*)*.

## Results and discussion

I applied the presented algorithm (Eq 1) to the GFP studied in [5]. Table 1 shows the comparison of the approximate [5] and the exact (this paper) increment volumes for nucleotide neighborhood up to 10 nucleotide substitutions away from the wild-type GFP sequence (238 amino acid residues; in the Table 1 first Met is considered constant, and the remaining 237 amino acid residues produce the presented diversity). The ratio of the numbers obtained by the exact and approximate algorithms increases with the number of nucleotide substitutions (Table 1). In the neighborhood of one to ten nucleotide substitutions, the results differ, at most, by a factor of 4.

**Table 1:** Comparison of approximate [5] and exact (this paper) number of possible amino acid sequences of GFP.

Number of nucleotide mutations from the wildtype	Number of possible amino acid sequences [5]	Number of possible amino acid sequences (this paper)	Ratio
1	1233	1424	1.2
2	759528	1011954	1.3
3	3.1 x 10^8^	4.8 x 10^8^	1.5
4	9.6 x 10^10^	1.7 x 10^11^	1.8
5	2.4 x 10^13^	4.8 x 10^13^	2.0
6	4.8 x 10^15^	1.1 x 10^16^	2.3
7	8.5 x 10^17^	2.3 x 10^18^	2.7
8	1.3 x 10^20^	4.0 x 10^20^	3.1
9	1.8 x 10^22^	6.2 x 10^22^	3.4
10	2.2 x 10^24^	8.7 x 10^24^	4.0

This difference may appear large. However, the size of protein sequence space is astronomically huge, so when dealing with such large numbers, the estimates can be acceptable if they deviate from the exact numbers by some small number of orders of magnitude [7]. Thus, from this point of view, I can conclude that the estimate made by Sarkisyan *et al*. is reasonably accurate.

Such loose requirement for the accuracy does not influence the fundamental significance of the algorithm, but diminishes its practical importance. If a rough estimate of the accessible sequence space size is close to the accurate calculation, the exact calculation may not be so important. However, the validity of the rough estimate itself was confirmed by the algorithm presented in this study. Furthermore, the exact calculation of the protein sequence space can become important in the future studies of protein evolution. When we learn more about decease-related mutations and come closer to the ultimate goal of molecular evolution—prediction of phenotype by genotype—it will be interesting to estimate more accurately the functional fraction of protein and nucleotide sequence spaces.

The presented algorithm somehow complements the exact translation from nucleotide to protein sequence for individual genes discovered more than 50 years ago. The algorithm calculates the similar exact correspondence but between the volumes of the nucleotide and protein sequence spaces. The result of the algorithm should not depend on the order of codons, which is similar to some machine-learning techniques where a protein sequence is reformatted into the vector of features; however, sometimes pseudo-techniques are used to save the order information [8,9].

The efficiency of the suggested algorithm was possible due to the power of a dynamic programming approach. Dynamic programming can solve optimization tasks of combinatorial nature, which otherwise could take astronomically long time [6]. The most famous dynamic programming algorithms in biology are the Needleman-Wunsch [10] and Smith-Waterman [11] algorithms for finding the optimal global and local alignments between two sequences. A dynamic programming approach can be used to calculate not only the optimal solution, but also some integral characteristics of the system, such as the volume of protein sequence space regions considered here. The generalization of the dynamic programming approach for diverse types of tasks was systematically presented in [12].

Note that the dynamic programming approach is not applicable (at least, directly) to some related questions. An example is the calculation of the volume of the protein sequence space accessible for a protein sequence by *exactly p* nucleotide substitutions in the protein-coding gene. The difficulty here arises because some sequences can be counted several times. For example, consider the dipeptide sequence SS coded by the nucleotide sequence UCG UCG. It can change to the dipeptide sequence LR after four nucleotide substitutions at least in two different ways (Fig 1a and 1b, left): one substitution in the first codon and three in the second, producing the sequence UUG AGA, or, alternatively, two substitutions in the first codon and two in the second, producing CUG AGG. It is not trivial to understand whether this task can be solved accurately and effectively.

## Conclusions

To summarize, I proposed the efficient and accurate algorithm to calculate the volume of protein sequence space accessible to a given protein sequence as a function of the number of nucleotide substitutions in its gene. I compared the exact (this paper) numbers with approximated by Sarkisyan *et al*. [5], and found that the relative error of the approximate solution increases with the number of considered nucleotide substitutions. However, the deviation is not huge: exact and approximate solutions differ, on average, by only the factor of 4 at ten nucleotide substitutions, and, therefore, approximate solution can be used for order of magnitude estimations. The implementation of the algorithm in PERL is available as Supplementary information.

## Supporting information

S1 File Implementation of the algorithm. The implementation of the algorithm written in PERL programming language. It requires that a file with genetic code tables is placed in the same directory (it is provided as the other additional file, see below). The program takes as a command line argument either the protein coding sequence or the file containing the protein-coding sequence in FASTA format. Auxiliary file with genetic code tables. The file contains genetic code tables needed for work of the PERL program. The file was taken from http://www.bioinformatics.org/jambw/2/3/TranslationTables.html on 31/05/2016, and table delimiters (" = = = = Table X = = = = " and " = = = = = = = = = = = = = = = = = ") were added before and after each table. The file consists of several sections. Each section includes: the information the name of a genetic code table, optional commentaries, the genetic code table itself embraced within lines containing equal signs, and optional commentaries.
(ZIP)

S2 File Time and space complexity. The consideration of time and space complexity of the algorithm.
(PDF)
