# Supplementary material for: Exact correspondence between walk in nucleotide and protein sequence spaces
Source: PLoS One. 2017 Aug 11;12(8):e0182525. doi: 10.1371/journal.pone.0182525 (PMC5553642; doi:10.1371/journal.pone.0182525)
Supplement: S2 File — The consideration of time and space complexity of the algorithm. (PDF) [file pone.0182525.s002.pdf]

Supplementary material to the paper Ivankov DN, PLoS ONE "Exact correspondence between walk in nucleotide and protein sequence spaces"

Dmitry N. Ivankov

June 12, 2017

Let us calculate the time and space required for the work of the algorithm described in the paper. Let R be the number of codons in nucleotide sequence, and the p is the number of nucleotide substitutions allowed. Note that $p \leq 3R$.
The whole graph similar to that described in the Fig.2 dissects into the 'linearly' increasing part and 'constant' part. In the linearly increasing part the number of nodes for each codon increases by three at every codon, while in the constant part the number of nodes for each codon is constant.

1. Let us consider the linearly increasing part. Let's assume that the number of codons in the linearly increasing part is k, so that, starting from k (inclusively) the amount of the nodes is constant till the end. The number of nodes in the beginning is 1, after one codon is 4, after $k-1$ is $1+3(k-1)$. So, the number of nodes for the linearly increasing part is

$$V_1 = 1 + 4 + ... + (1 + 3(k-1)) \quad (1)$$

$$V_1 = \frac{1 + (1 + 3(k-1))}{2}k \quad (2)$$

$$V_1 = \frac{(3k-1)k}{2} \quad (3)$$

Clearly,

$$k = \lceil \frac{p}{3} \rceil \quad (4)$$

That's why

$$V_1 = \frac{1}{2}\lceil\frac{p}{3}\rceil(3\lceil\frac{p}{3}\rceil - 1) \tag{5}$$

And

$$V_1 = O(p^2) \tag{6}$$

Now let us calculate the number of multiplications n_{m1} in the linearly increasing part:
For each node in the linearly increasing part it equals to the number of starting edges:

$$\begin{aligned} n_{m1} &= V_1 \times 4 = &(7)\\ &= 2k(3k-1) = &(8)\\ &= 2\lceil\frac{p}{3}\rceil(3\lceil\frac{p}{3}\rceil - 1) &(9)\end{aligned}$$

The number of multiplications is approximately the same for different p:

$$n_{m1} = \begin{cases} 2\lceil\frac{p}{3}\rceil(3\lceil\frac{p}{3}\rceil - 1), \text{ if } 3|p \\ 2\lceil\frac{p}{3}\rceil(3\lceil\frac{p}{3}\rceil - 1) - 1, \text{ if } 3|(p+1) \\ 2\lceil\frac{p}{3}\rceil(3\lceil\frac{p}{3}\rceil - 1) - 3, \text{ if } 3|(p+2) \end{cases} \tag{10}$$

Overall,

$$n_{m1} = O(p^2) \tag{11}$$

2. Now let us consider the constant part. Here after any codon, the number of nodes is p. The number of multiplications after each node equals to the number of transitions. The number of transitions corresponding to insertion of zero substitution in a codon is p, of one substitution $p-1$, for two substitutions $p-2$, for three substitutions $p-3$. Overall, the number of different possible mutations after a codon is $4p-6$. The number of codons in a constant part, after which we have transitions equals $(R-k)$. So, the overall number of transitions (and, in turn, the number of multiplications) is:

$$\begin{aligned} n_{m2} &= (R-k)(4p-6) = &(12)\\ &= (R - \lceil\frac{p}{3}\rceil)(4p-6) &(13)\\ & &(14)\end{aligned}$$

That is,

$$n_{m2} = O(pR) \tag{15}$$

The number of nodes in a constant part is

$$V_2 = (R - k + 1)p = \tag{16}$$

$$= (R - \lceil \frac{p}{3} \rceil + 1)p \tag{17}$$

That is,

$$V_2 = O(pR) \tag{18}$$

3. The number of summations at each node in the graph is not higher than four. So,

$$n_{sum} = 2\lceil \frac{p}{3} \rceil (3\lceil \frac{p}{3} \rceil - 1)+ \tag{19}$$

$$+ 4(R - \lceil \frac{p}{3} \rceil + 1)p \tag{20}$$

Or,

$$n_{sum} = O(p^2) + O(pR) \tag{21}$$

4. To summarize, the overall time complexity is

$$time = O(p^2) + O(pR) \tag{22}$$

For the small number of p this reduces to

$$time = O(pR) \tag{23}$$

At worst, $p = 3R$, and

$$time = O(R^2) \tag{24}$$

5. The space complexity. The space required for the algorithm is proportional to the number of nodes. This means that

$$space = O(p^2) + O(pR) \tag{25}$$

(26)

At small p

$$space = O(pR) \tag{27}$$

At worst,

$$space = O(R^2) \tag{28}$$
